# Supplementary material for: Low-Speed Clinorotation of Brachypodium distachyon and Arabidopsis thaliana Seedlings Triggers Root Tip Curvatures That Are Reminiscent of Gravitropism
Source: Int J Mol Sci. 2023 Jan 12;24(2):1540. doi: 10.3390/ijms24021540 (PMC9861679; doi:10.3390/ijms24021540)
Supplement: Supplementary file 1 [file ijms-24-01540-s001.zip › ijms-2006107-supplementary.pdf]

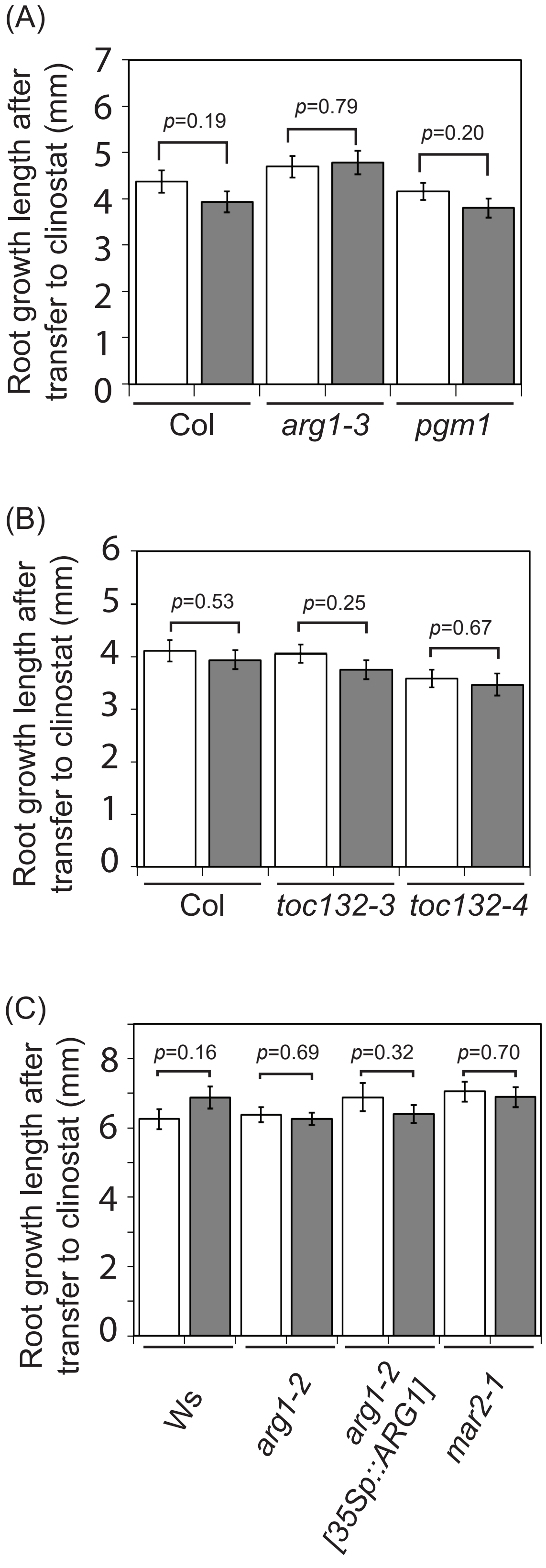

Supplementary Figure S1

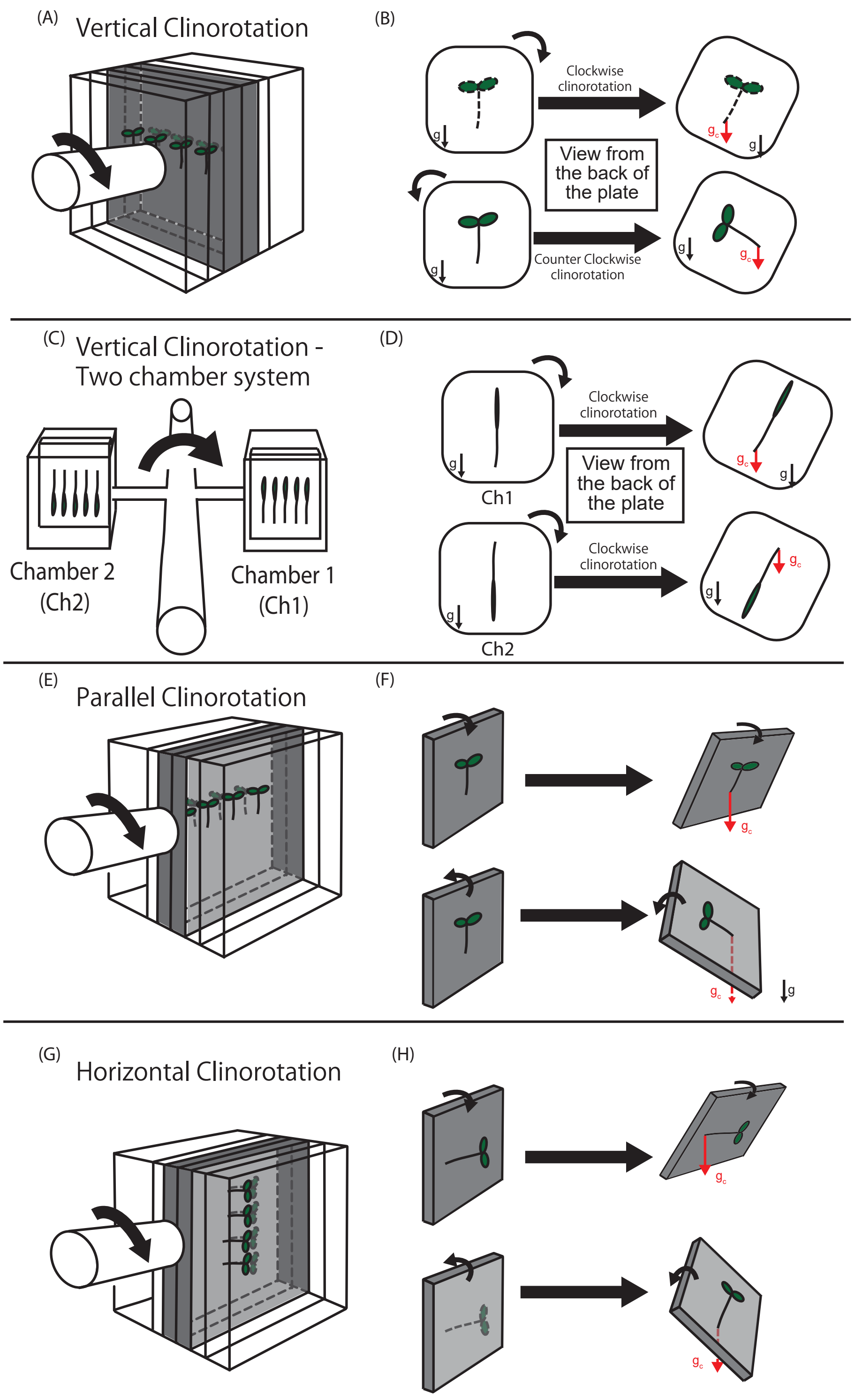

Supplementary Figure S2

(A) Two chamber clinostat system

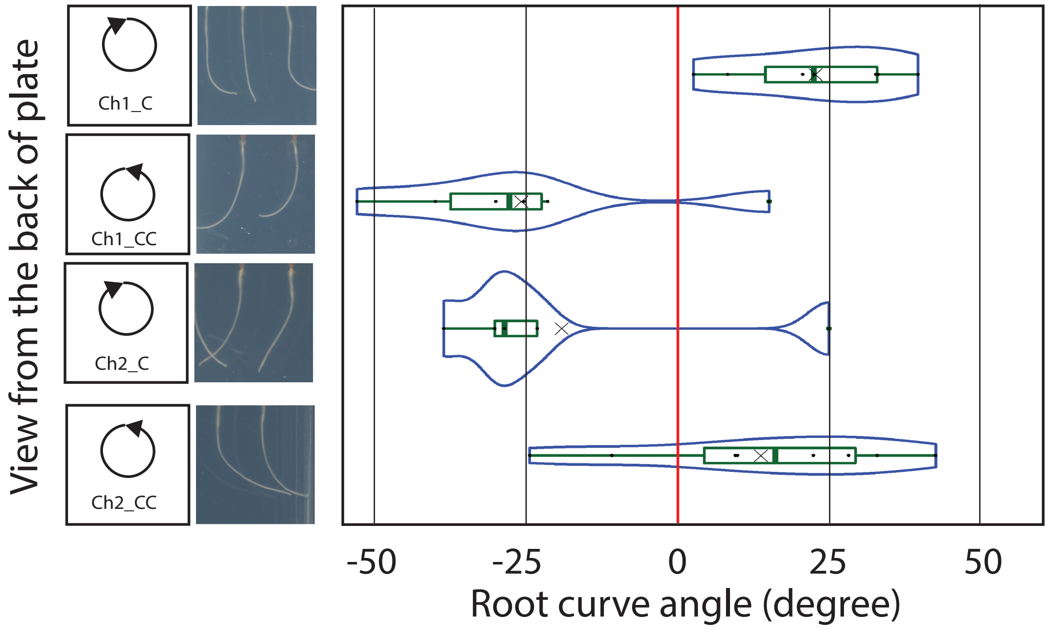

(B) Parallel Clinorotation

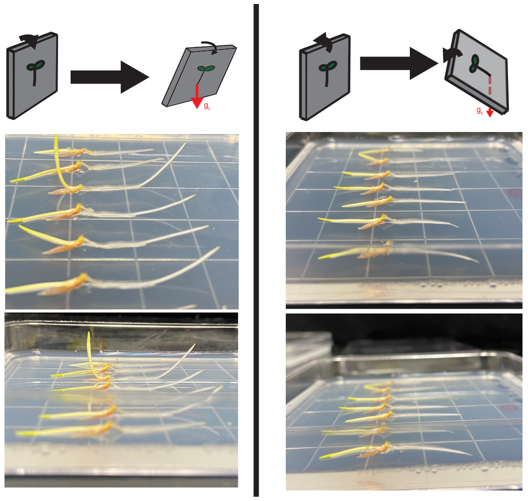

(C) Horizontal Clinorotation

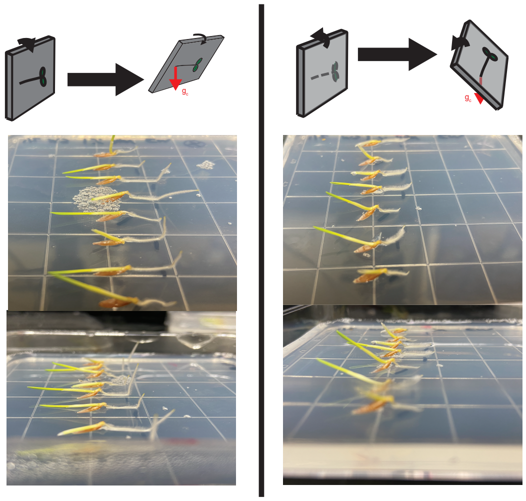

Supplementary Figure S3

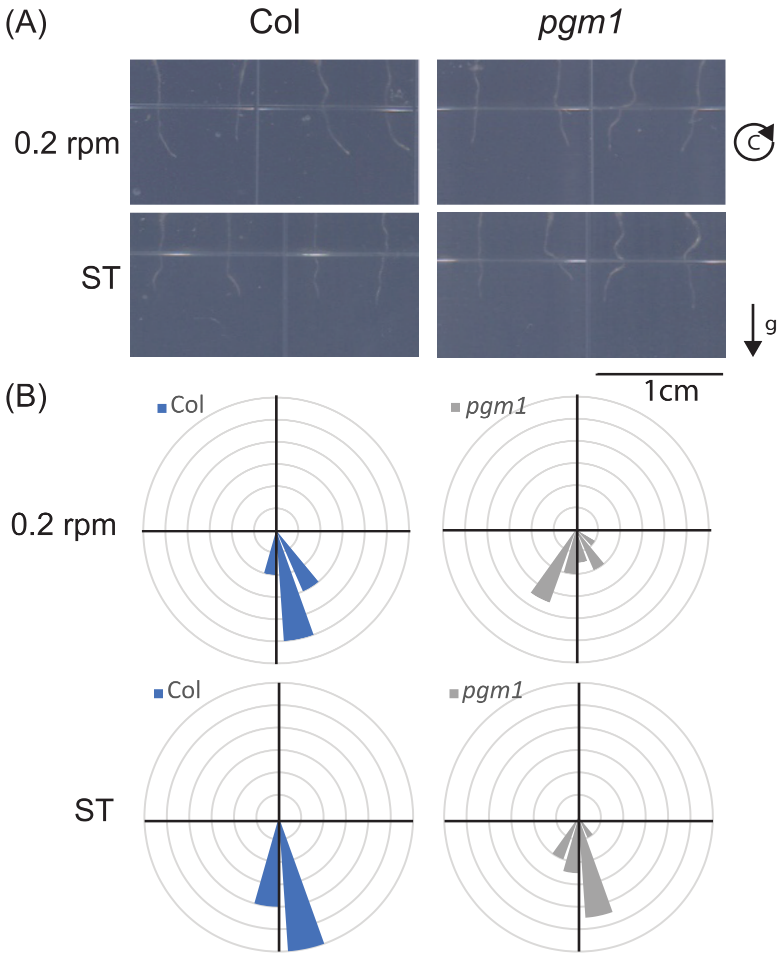

Supplementary Figure S4

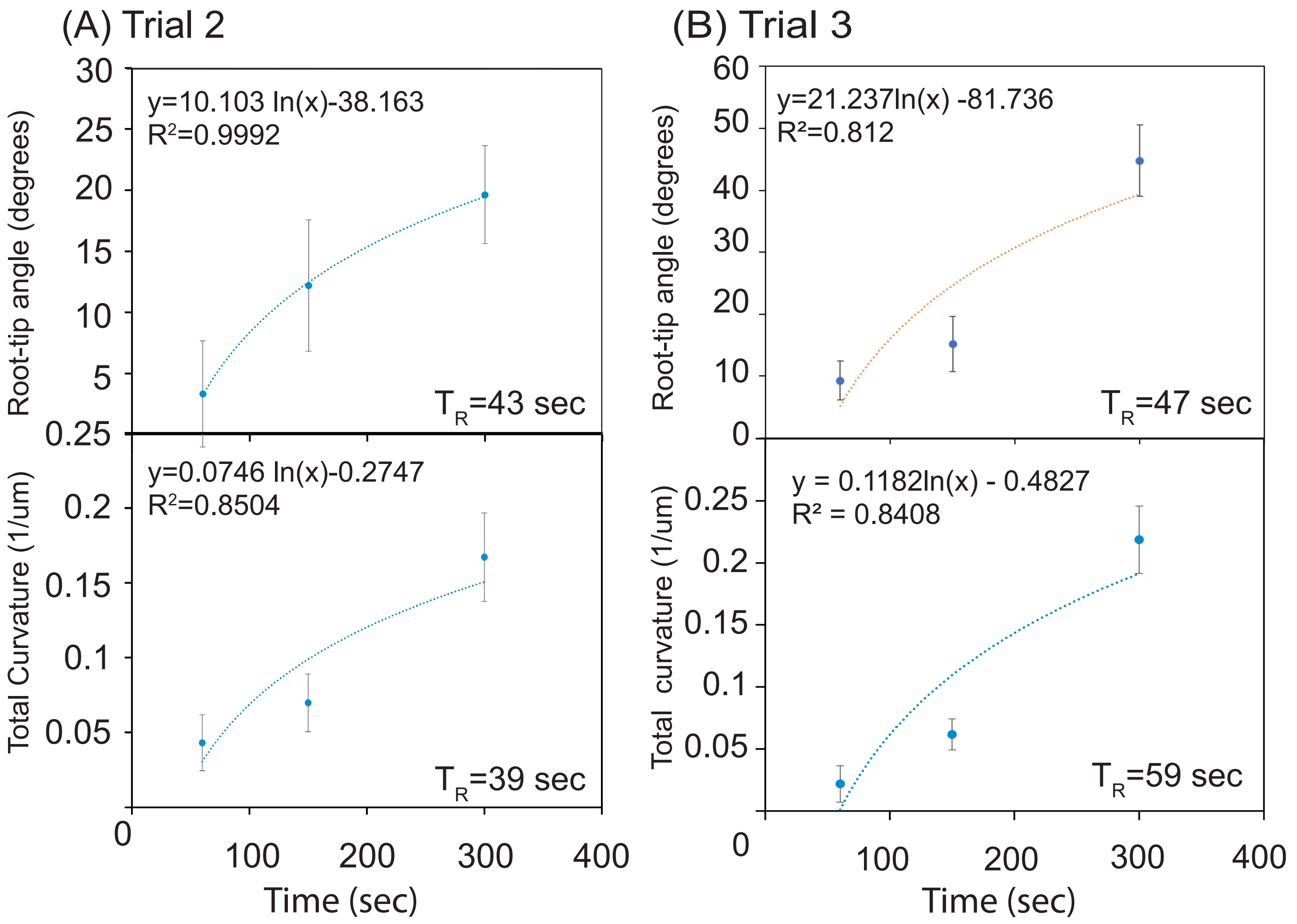

Supplementary Figure S5

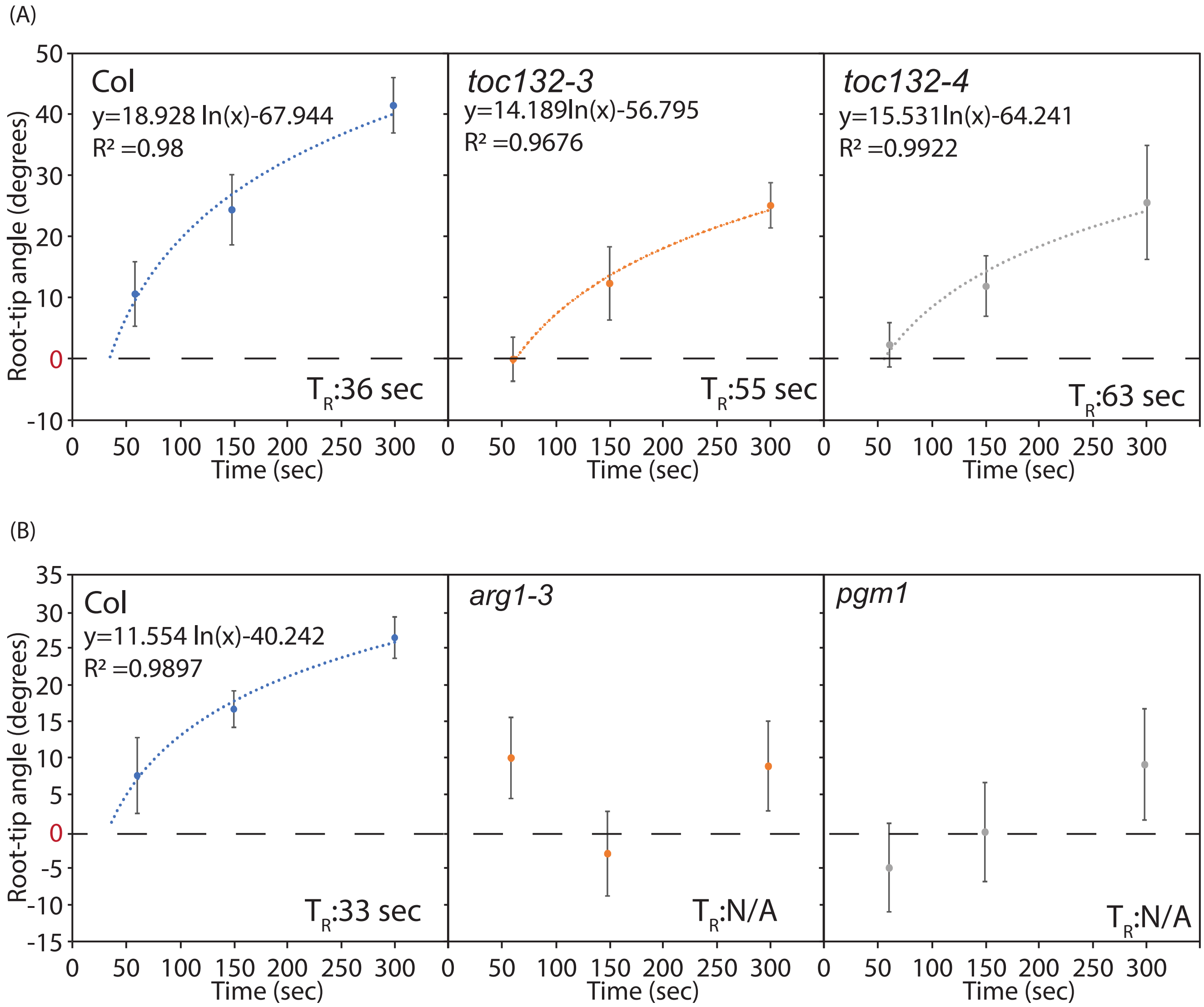

Supplementary Figure S6

(A)

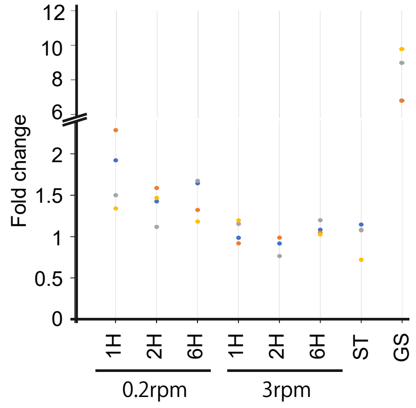

(B)

		Mean	Stdev	Sample size	*p-value of the t-test*						
						0.2rpm			3rpm		
					GS	1H	2H	6H	1H	2H	6H
	ST	1.01	0.19	4	9.2E-05	0.018	0.031	0.028	0.645	0.402	0.465
	GS	8.09	1.53	4		2.1E-04	1.3E-04	1.4E-04	9.4E-05	5.0E-04	9.5E-05
0.2rpm	1H	1.76	0.43	4			0.175	0.258	0.021	0.020	0.021
0.2rpm	2H	1.40	0.20	4				0.032	0.011	0.028	0.031
0.2rpm	6H	1.46	0.24	4					0.030	0.014	0.028
3rpm	1H	1.06	0.13	4						0.130	0.762
3rpm	2H	0.89	0.11	3							0.039
3rpm	6H	1.09	0.08	4							

Supplementary Figure S7

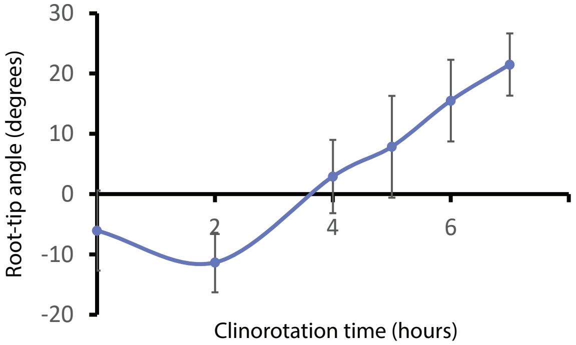

Suppementary Figure S8
